# Shen-Hong-Tong-Luo Formula Attenuates Macrophage Inflammation and Lipid Accumulation through the Activation of the PPAR-*γ*/LXR-*α*/ABCA1 Pathway

**DOI:** 10.1155/2020/3426925

**Published:** 2020-10-05

**Authors:** Zepeng Zhang, Lu Zhai, Jing Lu, Sanmiao Sun, Dandan Wang, Daqing Zhao, Liwei Sun, Weimin Zhao, Xiangyan Li, Ying Chen

**Affiliations:** ^1^Research Center of Traditional Chinese Medicine, The Affiliated Hospital to Changchun University of Chinese Medicine, Changchun, Jilin, China; ^2^Jilin Provincial Key Laboratory of Biomacromolecules of Chinese Medicine, Changchun University of Chinese Medicine, Changchun, Jilin, China; ^3^College of Traditional Chinese Medicine, Changchun University of Chinese Medicine, Changchun, Jilin, China; ^4^Jilin Ginseng Academy, Changchun University of Chinese Medicine, Changchun, Jilin, China; ^5^Center of Preventive Treatment of Disease, The Affiliated Hospital to Changchun University of Chinese Medicine, Changchun, Jilin, China; ^6^Department of Cardiology, The Affiliated Hospital to Changchun University of Chinese Medicine, Changchun, Jilin, China

## Abstract

Atherosclerosis (AS) is the killer of human health and longevity, which is majorly caused by oxidized lipoproteins that attack macrophages in the endarterium. The Shen-Hong-Tong-Luo (SHTL) formula has shown great clinical efficacy and vascular protective effect for over 30 years in China, to attenuate AS progression. However, its pharmacological mechanism needs more investigation. In this study, we first investigated the chemical composition of SHTL by fingerprint analysis using high-performance liquid chromatography. In primary mouse peritoneal macrophages induced by lipopolysaccharide (LPS), we found that SHTL pretreatment suppressed reactive oxygen species accumulation and reversed the increases of the inflammatory factors, TNF-*α* and IL-6. Moreover, lipid accumulation induced by oxidized low-density lipoprotein (Ox-LDL) in macrophages was inhibited by SHTL. Additionally, network pharmacology was used to predict the potential targets of SHTL as the PPAR-*γ*/LXR-*α*/ABCA1 signaling pathway, which was validated in macrophages and ApoE^−/−^ mice by histopathological staining, qPCR, and Western blot analysis. Importantly, the protective effect of SHTL in the LPS- and Ox-LDL-induced macrophages against inflammation and lipid accumulation was attenuated by GW9662, a PPAR-*γ* antagonist, which confirmed the prediction results of network pharmacology. In summary, these results indicated that SHTL pretreatment reduced inflammation and lipid accumulation of macrophages by activating the PPAR-*γ*/LXR-*α*/ABCA1 pathway, which may provide a new insight into the mechanism of SHTL in the suppression of AS progression.

## 1. Introduction

Atherosclerosis (AS) is a disease with narrowing of the arteries due to plaque accumulation, resulting in cardiac disorders, stroke, and vascular diseases with a high mortality rate worldwide [1]. Most studies have shown the importance of oxidized lipoprotein accumulation and inflammatory response in the pathogenesis of AS. Thus, the interventions targeting these pathogenic processes might be effective therapeutic strategies for AS treatment [2, 3]. Macrophages are the driving force in all stages of atherosclerosis [4], which can internalize oxidized low-density lipoprotein (Ox-LDL) to maintain the lipid balance in the blood vessels. However, macrophages usually lose their compensatory capacity and elicit local inflammation, causing themselves to become foam cells, which are the main pathogenic factors in AS [5, 6]. Collectively, the maintenance for macrophage homeostasis is the potential strategy to slow down the progression of AS [7].

Peroxisome proliferator-activated receptors (PPARs) are important regulators in many pathological processes, including plasma lipoprotein balance, foam cell formation, inflammatory response, and plaque stability [8]. Potential crosstalk has been identified between PPAR and liver X receptors (LXRs) for the prevention and treatment of AS [9]. PPAR-*γ* can induce cholesterol efflux from macrophages via the induction of LXRs [10, 11]. The LXRs can regulate cholesterol removal by the induction of plasma membrane transporters, such as ATP-binding cassette subfamily A member 1 (ABCA1) and subfamily G member 1 (ABCG1) [12]. Meanwhile, recent studies have revealed that the activation of PPAR-*γ* can reduce the expression of inflammatory factors in the aortic root, thus inhibiting the development of AS [13]. Therefore, the PPAR-*γ*/LXR-*α* pathway could be a potential therapeutic target in regulating the progression of AS [14].

For centuries, traditional Chinese medicine (TCM) has played an irreplaceable role in Chinese healthcare [15]. In Chinese medicine, the disease characteristics of AS have already been recorded and called *Xiong Bi* with common symptoms, like Qi deficiency, phlegm stagnation, and blood stagnation, which are used to guide TCM doctors for AS diagnosis and treatment [16–18]. According to the principles of TCM above, the Shen-Hong-Tong-Luo (SHTL) formula was developed and used to treat the patients with AS by tonifying Qi and invigorating blood circulation for over thirty years in the Affiliated Hospital to Changchun University of Chinese Medicine by decreasing the incidence of secondary terminal events and total angina pectoris score [19, 20]. SHTL contains eight herbs, including *Panax ginseng C*.*A*. *Mey*, *Salvia miltiorrhiza Bunge*, *Rhodiola crenulata* (*Hook*.*f*. *& Thomson*) *H*. *Ohba*, *Lonicera japonica Thunb*, *Paeonia anomala* subsp. *veitchii* (*Lynch*) *D*.*Y*.*Hong & K*.*Y*.*Pan*, *Trichosanthes kirilowii Maxim*, *Angelica sinensis* (*Oliv*.) *Diels*, and *Dalbergia odorifera T*.*C*.*Chen*. However, the molecular mechanism of SHTL against AS progression remains unclear. Here, we first elucidated the protective effects of SHTL against inflammation and lipid accumulation in the LPS- or Ox-LDL-induced macrophages. Next, the potential target of SHTL, the PPAR-*γ*/LXR-*α*/ABCA1 signaling pathway, was predicted by network pharmacology and validated by a series of experiments using a PPAR-*γ* antagonist. This study may provide a new insight on the therapeutic effects and molecular mechanism of SHTL against AS progression.

## 2. Materials and Methods

### 2.1. Reagents

Ox-LDL and DiI-Ox-LDL were purchased from Yiyuan Biotechnology (Guangzhou, China). Antibodies against PPAR-*γ* (57 kDa, ab59256), LXR-*α* (50 kDa, ab41902), and ABCA1 (254 kDa, ab18180) were purchased from Abcam (Cambridge, MA, USA). *β*-Actin (42 kDa, #3700) was purchased from Cell Signaling Technology (Beverly, MA, USA). GW9662 was obtained from MedChemExpress (Monmouth Junction, NJ, USA).

### 2.2. Preparation of SHTL

The 8 herbs present in SHTL were purchased from the Department of Pharmacy, the Affiliated Hospital to Changchun University of Chinese Medicine (Jilin, China). The composition of SHTL at a weight ratio of 4 : 5 : 3 : 3 : 3 : 4 : 4 : 2 is presented in Table 1 and was deposited to the Research Center of Traditional Chinese Medicine, the Affiliated Hospital to Changchun University of Chinese Medicine. According to the standard procedure from *Chinese Pharmacopoeia* (2015 edition), 280 g of herb mixture was extracted in 300 mL of distilled water at 100°C for 30 min. The procedure was repeated two times to obtain the aqueous extract. The aqueous extract was filtered and centrifuged; and the supernatant was dried under vacuum to produce a powder with a yield of 15.71% [21].

### 2.3. Quality Control Analysis

Based on previous reports, 10 different batches of SHTL powders were separated using a ZORBAX SB-C18 column (4.6 × 250 mm, 5 *μ*m, Agilent, Santa Clara, CA, USA) and analyzed for chemical fingerprints using high-performance liquid chromatography (HPLC, Shimadzu Corp., Nakagyo-ku, Kyoto, Japan) coupled with a diode array detector (DAD) (Shimadzu Corp.) [21]. Acetonitrile in water (solvent A) and 0.4% phosphoric acid in water (solvent B) constituted the mobile phase (Supplementary Table 1). The flow rate was 1.0 mL/min at 25°C, and the detection wavelength was set at 203 nm. Salidroside, chlorogenic acid, paeoniflorin, ferulic acid, luteoloside, ginsenoside Rg1, and luteolin were used as controls to analyze the retention time (Shanghai Yuanye Biotechnology Co., Ltd.; Shanghai, China).

### 2.4. Animal Study

All animal protocols adopted in the present study were approved by the Experimental Animal Administration Committee of Changchun University of Chinese Medicine (Approval No. 20190048), which follows the guidelines established by the US National Institutes of Health. After adaptive feeding for 1 week, ApoE^−/−^ mice from Beijing Huafukang Biotechnology Co., Ltd., were fed with high-fat diet (HFD, 40 kcal% fat, 1.25% cholesterol, 0.5% cholic acid, D12109, Research Diets, Inc., NJ, USA) for 6 weeks, then orally administrated with SHTL, and received HFD for 8 weeks. All animals are kept in a specific pathogen-free environment.

### 2.5. Analysis of the Atherosclerotic Plaque Area

The heart and aorta were extracted, formalin-fixed, paraffin-embedded to make tissue blocks, which were cut into 6 *μ*M sections. Tissue sections were deparaffinized, rehydrated, and covered with 3% H_2_O_2_ for 10 min. After blocking with BSA, slides were incubated with primary antibodies, PPAR-*γ*, LXR-*α*, and ABCA1 for 1 h at 37°C and with corresponding secondary antibodies for an additional 1 h. Antigenic sites were visualized by the addition of DAB and counterstained with hematoxylin. *En face* aorta Oil red O staining was performed to evaluate lipid and plaque accumulation in the aorta following the protocol published before [22]. Cryocut cross-sections were washed twice with ultrapure water and stained with 0.3% Oil red O (Sigma-Aldrich, USA) in 60% isopropanol for 10 min, washed with 75% isopropanol for 1 min, and then counterstained with hematoxylin (Sigma-Aldrich) for 1 min. After washing with ultrapure water for 3 min, cross-sections were covered with glycerogelatin. The positively stained areas were determined using Image-Pro Plus 5.0 software.

### 2.6. Cell Culture and Model Establishment

Briefly, 8-week-old mice received intraperitoneal injections of 4% Brewer thioglycollate medium (Difco, Detroit, MI, USA) to produce peritoneal macrophages. After 3 days, the peritoneal cells were isolated in ice-cold PBS, centrifuged, and washed with lysis buffer to remove red blood cells (Beyotime Biotechnology, Shanghai, China). Next, cells were seeded in culture dishes for 2 h followed by washing with PBS to remove floating cells. After 24 h incubation, adherent cells were cultured in RPMI 1640 medium (Gibco, New York, NY, USA) supplemented with 10% fetal bovine serum (FBS, Clark Bioscience, Claymont, DE, USA), 100 units/mL penicillin, and 100 *μ*g/mL streptomycin (Biosharp, Hefei, China) at 37°C in a humidified atmosphere with 5% CO_2_ [23]. THP-1 human monocytes were cultured in RPMI 1640 medium with phorbol 12-myristate 13-acetate (PMA, 150 nM) for 24 h to differentiate into adherent macrophages [24]. Peritoneal macrophages and differentiated THP-1 cells were incubated with LPS (100 ng/mL; Sigma-Aldrich, St. Louis, MO, USA) for 8 h or Ox-LDL (40 *μ*g/mL) for 24 h to establish cell models of inflammation or lipid accumulation for further experimentation.

### 2.7. Cell Viability Assay

Cells were seeded into 96-well cell culture plates and treated with SHTL for 48 h. After adding MTT (0.5 mg/mL), the formazan crystals were dissolved with 150 *μ*L of DMSO, and the absorbance was measured at 490 nm using a microplate reader (Infinite M200 PRO, Tecan, ZH, Switzerland) [25].

### 2.8. Reactive Oxygen Species (ROS) Measurement

Cells were treated with various concentrations of SHTL for 24 h, prior to incubation with 100 ng/mL LPS for 8 h. Next, they were equilibrated in carboxy-H2DCFDA (10 *μ*M, Beyotime Biotechnology, Shanghai, China) in culture medium for another 20 min at 37°C. Fluorescence intensity was analyzed by flow cytometry (BD Biosciences, San Jose, CA, USA) [26].

### 2.9. Enzyme-Linked Immunosorbent Assay (ELISA)

After incubation with SHTL, LPS, and GW9662, the supernatants of macrophages from each group were collected. The levels of the proinflammatory factors TNF-*α* and IL-6 in the supernatants were detected using the mouse TNF-*α* (88-7324-22) and IL-6 (88-7064-22) ELISA kits (Invitrogen, Carlsbad, CA, USA) [27].

### 2.10. DiI-Ox-LDL Uptake Assay

To assess lipid accumulation in macrophages, different groups of cells were serum-starved for 8 h, followed by exposure to 50 *μ*g/mL of DiI-Ox-LDL for 4 h. Then, the samples were washed with PBS and analyzed using a flow cytometry (BD Biosciences); the images were collected and analyzed using a high-content analysis system (Perkin Elmer, Massachusetts, USA).

### 2.11. Cholesterol Efflux Fluorometric Assay

To assess cholesterol efflux in macrophages, this assay was conducted as previously described [28]. Briefly, after SHTL pretreatment for 24 h, macrophages were incubated with fluorescently labeled cholesterol for 4 h in a humidified cell culture incubator and then incubated in a medium containing different interventions for another 4 h. At the end of incubation, we transferred the supernatants of each well (including control wells) to a white 96-well plate (with opaque, flat-bottom wells) and measured the fluorescence (Ex/Em = 485/523 nm) in the endpoint mode. The adherent cells were solubilized by cell lysis buffer to measure the fluorescence (Ex/Em = 482/515 nm): cholesterol efflux% = fluorescence intensity of the media/fluorescence intensity of the cell lysate + media∗100.

### 2.12. Quantitative Real-Time PCR (qPCR)

Total RNA from the macrophages treated under different conditions was extracted to determine the relative mRNA levels of PPAR-*γ*, LXR-*α*, ABCA1, IL-6, and TNF-*α* using the total RNA kit (Omega, Norcross, GA, USA). 1 *μ*g of RNA was reverse transcribed into cDNA using the iScript cDNA synthesis kit (Bio-Rad, USA). The assay was performed in a Bio-Rad CFX96 system, and the gene expression was normalized to *β*-actin and calculated using the 2^-*ΔΔ*Ct^ method [29]. The primer sequences are shown in Supplementary Table 2.

### 2.13. Western Blotting Analysis

Western blot analysis was conducted using a previously described method [30]. Cells were lysed using RIPA buffer, and protein concentration was quantified using a BCA protein assay kit (Beyotime Biotechnology). Equal amounts of protein (40 *μ*g) were separated using 8% or 12% SDS-PAGE and transferred onto polyvinylidene difluoride (PVDF) membrane. The membranes were incubated with specific primary antibodies (PPAR-*γ*, LXR-*α*, and ABCA1) overnight at 4°C after blocking with 5% nonfat milk, followed by incubation with appropriate secondary antibodies. The protein bands were visualized and analyzed using a chemiluminescent imaging system (FluorChem, ProteinSimple, San Jose, CA, USA).

### 2.14. Target Prediction of SHTL

Network pharmacology was conducted to identify the potential targets of the eight herbs in SHTL. The SHTL formula was input by the herb list (pinyin name) to the BATMAN-TCM database. The required parameters settings were score cutoff = 20, and the enrichment analyses of a group of protein targets are all based on the hypergeometric cumulative distribution test (for KEGG enrichment pathway analysis, we exclude those targets located in ≥10% KEGG pathways), and the multiple testing correction of the *P* value was based on the Benjamini-Hochberg correction method [31]. Data visualization of the herb components and the potential pathways for SHTL were obtained using Gephi 0.9.2 software and OmicShare Tools, respectively [32, 33]. Then, we screened the relevant compounds from SHTL with the following screening parameters: oral bioavailability ≥ 30%, drug likeness ≥ 18%, blood‐brain barrier ≥ −0.3%, and drug half‐life ≥ 4 h to dig out the core targets in the potential pathways. In the STRING database, the basic and analytic settings, including the meaning of network edges, text mining, experiments, and databases, were selected to analyze the potential target proteins from the above candidates [34]. Finally, the core potential targets of SHTL were validated using the prediction of disease targets by BATMAN-TCM database.

### 2.15. Statistical Analysis

Data from three independent experiments were expressed as the mean ± standard deviation and were studied using GraphPad Prism 7.0 (GraphPad Software, La Jolla, CA, USA). For multiple comparisons, data were subjected to one-way ANOVA (Turkey's post hoc) to determine statistical significance. For all the statistical tests, *P* < 0.05 was considered statistically significant.

## 3. Results

### 3.1. Quality Control Analysis of SHTL

**Figure 1:**
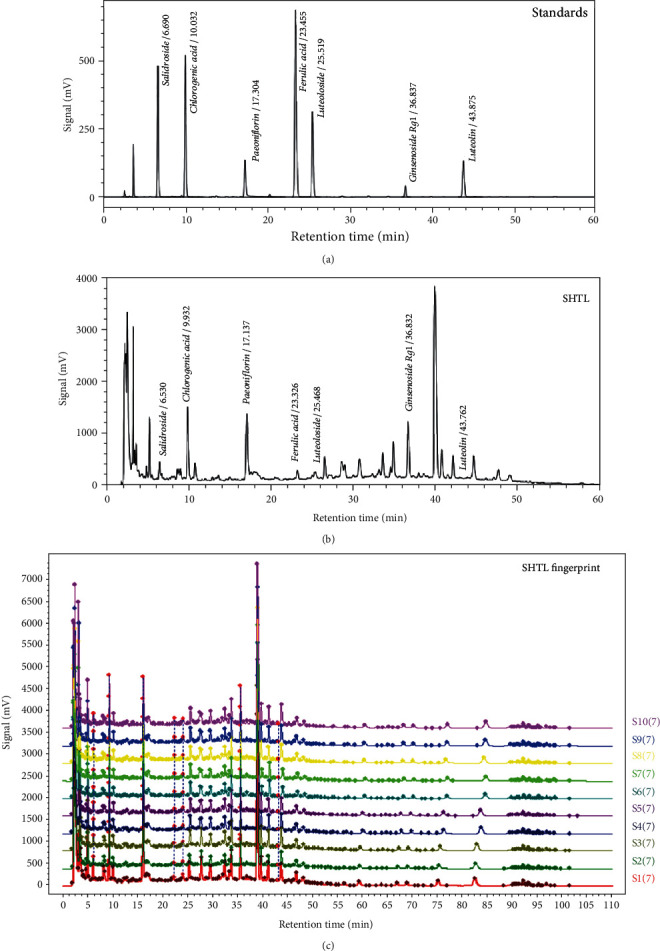
Quality control analysis of the SHTL formula. (a) HPLC chromatograms of mixed standards including salidroside, chlorogenic acid, paeoniflorin, ferulic acid, luteoloside, ginsenoside Rg1, and luteolin at 203 nm. (b) HPLC chromatogram of SHTL at 203 nm. (c) The reproducible HPLC fingerprints of 10 batches of SHTL (S1-S10), using the Chinese Medicine Chromatographic Fingerprint Similarity Evaluation System (2012 Edition).

To investigate the main components and the similarity between different batches of the SHTL formula, we investigated the chromatographic fingerprints of SHTL and standard compounds. As shown in Figures 1(a) and 1(b), twenty-five peaks of the SHTL extract were identified through the HPLC fingerprints. Seven peaks were identified by comparing the retention times with the standards. These included salidroside in *Rhodiola*, chlorogenic acid, luteoloside, luteolin in honeysuckle, paeoniflorin in *Paeoniae radix rubra*, ferulic acid in *Angelica*, and ginsenoside Rg1 in *Panax ginseng C*.*A*. *Mey* in SHTL (Figure 1(b)). Moreover, all the 10 batches of SHTL were found to be 98.4% to 99.9% similar, indicating that the SHTL formula was reproducible with good quality control (Figure 1(c)).

### 3.2. SHTL Inhibits Inflammation Response in LPS-Induced Macrophages

**Figure 2:**
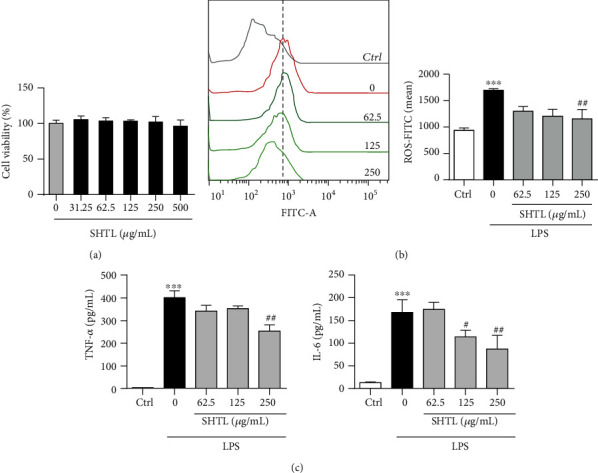
SHTL inhibits LPS-induced inflammatory response in mouse peritoneal macrophages. (a) After treatment with different doses of SHTL for 48 h, the dose-dependent cytotoxicity of SHTL in mouse peritoneal macrophages (MPMs) was evaluated by MTT assay. (b) SHTL pretreatment for 24 h inhibited the intracellular ROS accumulation in MPMs that was induced by LPS (100 ng/mL, 8 h), determined with DCFH-DA staining followed by flow cytometric analysis. The mean of DCFH-DA-FITC fluorescence is shown on the right. (c) After SHTL pretreatment for 24 h, the levels of TNF-*α* and IL-6 in the supernatants of MPMs were detected by ELISA kits. ^∗∗∗^*P* < 0.001 versus the Ctrl group; #*P* < 0.05 and *^##^P* < 0.01 versus the LPS group, *n* = 3.

To observe dose-dependent cytotoxic effects of SHTL in mouse peritoneal macrophages (MPMs), cell viability was measured. As shown in Figure 2(a), SHTL had no cytotoxic effect at concentrations below 500 *μ*g/mL on macrophages after 24 h of pretreatment. To further examine the anti-inflammatory effect of SHTL, MPMs were treated with different doses of SHTL for 24 h and incubated with LPS for 8 h. In the LPS-induced cell model, LPS incubation significantly increased the level of intracellular ROS, which was reduced by SHTL pretreatment (Figure 2(b)). Meanwhile, LPS increased the levels of TNF-*α* and IL-6 in the supernatants of MPMs to 400.71 pg/mL and 167.38 pg/mL, respectively. SHTL pretreatment for 24 h significantly attenuated the increase in expression of TNF-*α* and IL-6 that was induced by LPS (Figure 2(c)). These results indicated that SHTL pretreatment inhibited the inflammatory response induced by LPS in macrophages.

### 3.3. SHTL Reduces Lipid Accumulation in the Ox-LDL-Induced Macrophages

**Figure 3:**
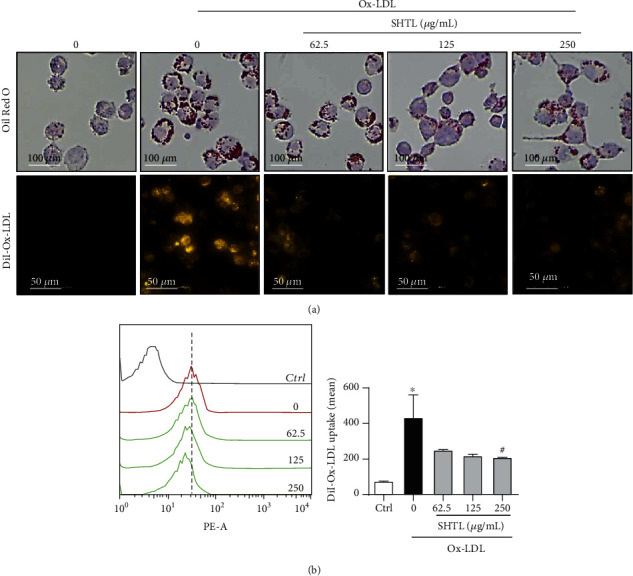
SHTL reduces Ox-LDL-induced cellular lipid accumulations in macrophages. (a) MPMs were stimulated without or with Ox-LDL (40 *μ*g/mL, 24 h) in the presence or absence of SHTL for 24 h. The dose-dependent lipid-reducing effect of SHTL pretreatment in MPMs was evaluated using the Oil red O (top) or DiI-Ox-LDL (bottom) stainings. Representative microscopic images (40x, scale bar = 50 *μ*m) are shown. (b) Pretreatment with SHTL for 24 h inhibited cellular lipid accumulation in THP-1-drived macrophages using flow cytometric analysis. The quantification of DiI-Ox-LDL-PE fluorescence is shown on the right. ^∗^*P* < 0.05 versus the Ctrl group; ^#^*P* < 0.05 versus the Ox-LDL group, *n* = 3.

Further, we performed Oil red O or DiI-Ox-LDL staining to evaluate the protective effect of SHTL against Ox-LDL-induced lipid accumulation in mouse peritoneal and THP-1-differentiated macrophages. As shown in Figure 3(a) (top), Oil red O staining showed that pretreatment with SHTL for 24 h lowered the Ox-LDL-induced cellular accumulation of lipid droplets in MPMs. Moreover, fluorescence microscopic analysis revealed that Ox-LDL stimulation increased DiI-Ox-LDL internalization, which was inhibited by SHTL pretreatment in a dose-dependent manner (Figure 3(a), bottom). Additionally, flow cytometric analysis showed that pretreatment with SHTL inhibited the uptake of DiI-Ox-LDL in THP-1-drived macrophages (Figure 3(b)). These results indicated that SHTL obviously reduced lipid accumulation in Ox-LDL-induced macrophages.

### 3.4. The Target Prediction of SHTL by Network Pharmacology

**Figure 4:**
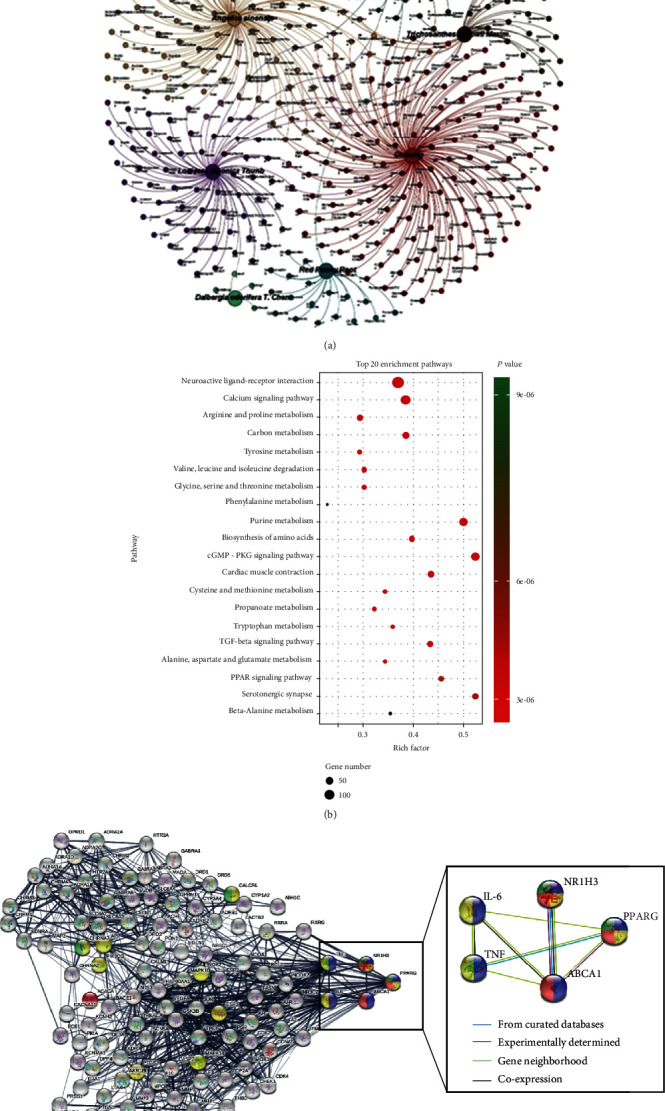
Potential targets of SHTL are predicted by network pharmacology. (a) The compound-herb network of SHTL was analyzed by network pharmacology. The bigger circles represent the herbs, and the smaller circles represent the compounds (b) Bubble diagram showed the top 20 enrichment pathways of SHTL. The pathway, gene number, and *P* value are shown. (c) The STRING analysis showed the network interaction of the PPAR signaling pathway. NR1H3 stands for LXR-*α* as its gene name. The colors of nodes represent different biological pathways: blue for the negative regulation of lipid storage response, and red for the lipid homeostasis. The line thickness indicates the strength of data support. The different colored lines between nodes represent the evidence that came from databases or experiments.

The molecular mechanism of SHTL involved in the reduction of inflammation and lipid accumulation was explored using BATMAN-TCM, to find the potential targets of the candidate compounds from SHTL. Based on the screening conditions, 447 candidate active compounds were identified in SHTL (Figure 4(a)) and are listed in Table 2. After GO enrichment pathway analysis, we found that the target pathways significantly involved in the mechanism of SHTL included the neuroactive ligand-receptor interaction; calcium signaling pathway; glycine, serine, and threonine metabolism; TGF-*β* signaling pathway; and PPAR signaling pathway (Supplementary Table 3). The top 20 enrichment pathways of SHTL are shown in Figure 4(b) and are also listed in Supplementary Table 3. Furthermore, the STRING database was used for digging out the core targets of active compounds in SHTL. As shown in Figure 4(c), 112 nodes and 841 edges were found and built the interactions of active compounds of SHTL and their targets, with an average local clustering coefficient 0.552 and the PPI enrichment *P* value < 1.0*e*-16. Among these nodes, the PPAR-*γ* pathway was the most interactive and participated in the negative regulation of lipid storage and inflammatory response and lipid homeostasis (Figure 4(c) and Supplementary Table 4). Meanwhile, SHTL showed a strong regulation and control function of PPAR-*γ* on NR1H3 (LXR-*α*), ABCA1, TNF-*α*, and IL-6, which were involved in the regulation of inflammatory response and lipid accumulation. Finally, the disease target network verified the central role of PPAR-*γ* in regulating AS, inflammation, cardiovascular disease, hypertension, and other diseases (Supplementary Figure S1). These results suggested that the PPAR-*γ* pathway may be the core potential target of SHTL for inhibiting the inflammatory response and lipid accumulation.

### 3.5. SHTL Activates the PPAR-*γ*/LXR-*α*/ABCA1 Pathway in the Ox-LDL-Induced Macrophages

**Figure 5:**
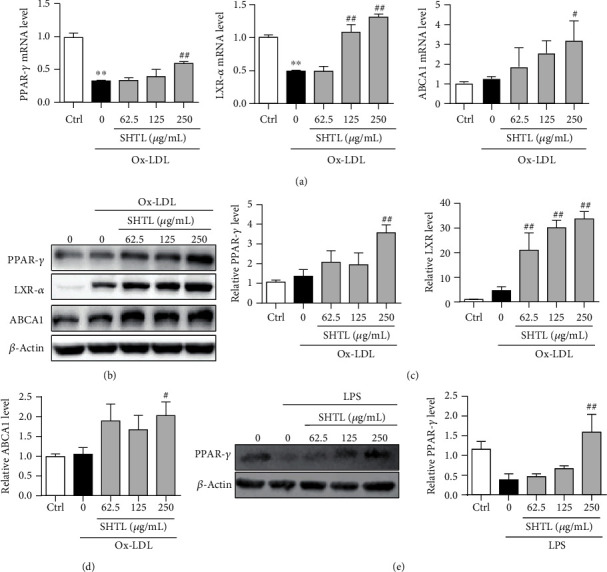
SHTL activates the PPAR-*γ*/LXR-*α*/ABCA1 pathway in the Ox-LDL-induced mouse peritoneal macrophages. (a) After SHTL and Ox-LDL incubation for 24 h, the mRNA levels of PPAR-*γ*, LXR-*α*, and ABCA1 in mouse peritoneal macrophages (MPMs) were determined by qPCR analysis after the normalization using *β*-actin. (b) MPMs were incubated with SHTL for 24 h prior to Ox-LDL incubation for 24 h. The whole cell lysates were subjected to electrophoresis in SDS-PAGE gels for Western blot analysis. *β*-Actin was the loading control. (c) Bar graph shows relative expression levels of these proteins from (b). (d, e) After SHTL incubation for 24 h before induced by LPS (8 h), the protein levels of PPAR-*γ* in MPMs were determined by Western blot analysis. *β*-Actin was the loading control. ^∗∗^*P* < 0.01 versus the Ctrl group; ^#^*P* < 0.05 and ^##^*P* < 0.01 versus the Ox-LDL or LPS group, *n* = 3.

Based on the results of enrichment analysis of network pharmacology, we further validated whether the protective effect of SHTL against inflammation and lipid accumulation was dependent on the PPAR-*γ* signaling pathway. The PPAR-*γ*/LXR-*α* signaling pathway is a negative regulator of inflammation and lipid metabolism, which also regulates ABCA1 expression for lipid homeostasis [35, 36]. qPCR and Western blot analyses detected the expression profiles of PPAR-*γ*, LXR-*α*, and ABCA1 in macrophages. We first found that SHTL pretreatment increased the mRNA levels of LXR-*α* and ABCA1 in untreated macrophages (Supplementary Figure S2A). In the Ox-LDL-induced macrophages, qPCR analysis showed that Ox-LDL inhibited the levels of LXR-*α* and ABCA1 in a time-dependent manner (Supplementary Figure S2B). Importantly, SHTL pretreatment for 24 h significantly increased the mRNA levels of PPAR-*γ*, LXR-*α*, and ABCA1 in a dose-dependent manner in the Ox-LDL-induced macrophages (Figure 5(a)). Moreover, SHTL pretreatment upregulated the protein levels of the PPAR-*γ*/LXR-*α*/ABAC1 pathway, compared with the Ox-LDL induction (Figures 5(b)–5(d)). In addition, we found that SHTL also could upregulate the protein level of PPAR-*γ* after LPS incubation in macrophages (Figure 5(e)). Therefore, these findings suggested that SHTL activated the PPAR-*γ*/LXR-*α*/ABAC1 pathway in Ox-LDL- and LPS-induced macrophages.

### 3.6. SHTL Decreased the Progression of Atherosclerotic Plaque and Regulates the PPAR-*γ*/LXR-*α*/ABAC1 Pathway in ApoE^−/−^ Mice

**Figure 6:**
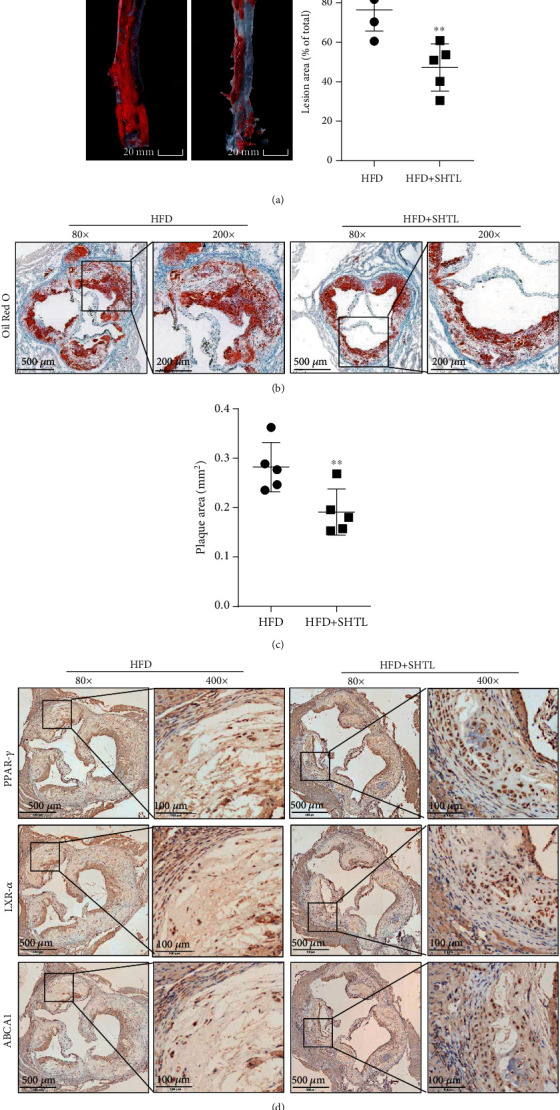
SHTL decreased progression of atherosclerotic plaque and regulated PPAR-*γ*/LXR-*α*/ABAC1 expression in ApoE^−/−^ mice. (a) Representative images of Oil red O staining of *en face* preparations of aortas and the quantification of the atherosclerotic surface area of the entire aorta (*n* = 5, scale bar = 20 mm). (b, c) Representative images and the quantification of aorta sections stained with Oil red O (*n* = 5, scale bar of 80x = 500 *μ*m, scale bar of 400x = 100 *μ*m). (d) Positive expression of PPAR-*γ*, LXR-*α*, and ABCA1 in aorta sections detected by immunohistochemical staining. Representative images with 80x and 400x are shown. (*n* = 5, scale bar of 80x = 500 *μ*m, scale bar of 400x = 100 *μ*m). ^∗∗^*P* < 0.01 versus the HFD group.

To further examine the antiatherosclerotic effect of SHTL, we collected the aortic tissues in ApoE^−/−^ mice after 6 weeks of high-fat diet and 8 weeks of SHTL intervention. Both *en face* aorta and cross-section Oil red O stainings showed that SHTL reduced the lesion area and atherosclerotic plaque area for about 30% (Figures 6(a)–6(c)). Meanwhile, immunohistochemical staining in Figure 6(d) showed that SHTL upregulated the protein expressions of PPAR-*γ*, LXR-*α*, and ABAC1 in plaque. The fatty degeneration and plaque volume of the SHTL group were significantly smaller than the HFD group (Figure 6(d)). These results proved that SHTL decreased the progression of atherosclerotic plaque by upregulating the PPAR-*γ*/LXR-*α*/ABAC1 pathway in ApoE^−/−^ mice.

### 3.7. SHTL Inhibits LPS-Induced Inflammatory Response in a PPAR-*γ*-Dependent Manner in Macrophages

**Figure 7:**
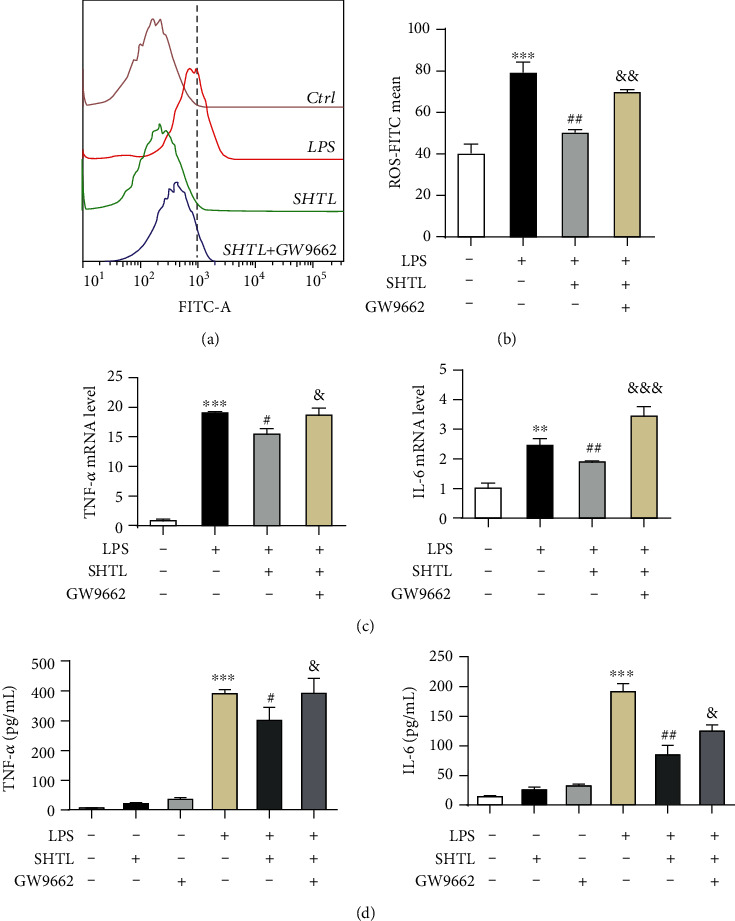
SHTL inhibits LPS-induced inflammatory response in a PPAR-*γ* dependent manner in mouse peritoneal macrophages. (a) MPMs were incubated with SHTL (125 *μ*g/mL) or GW9662 (PPAR-*γ* antagonist, 10 *μ*M) for 1 h, then exposed to LPS (100 ng/mL) for 8 h. Intracellular ROS levels in MPMs were evaluated using flow cytometry. (b) Bar graph shows fluorescence intensity of ROS content in different groups from (a). (c, d) After the pretreatment with SHTL and/or GW9662 along with the incubation with LPS, the relative mRNA levels of TNF-*α* and IL-6 in MPMs and the content of TNF-*α* and IL-6 in the culture supernatants were detected by qPCR and ELSIA kits, respectively. ^∗∗^*P* < 0.01 and ^∗∗∗^*P* < 0.001 versus the Ctrl group; ^#^*P* < 0.05 and ^##^*P* < 0.01 versus the LPS group; ^&^*P* < 0.05, ^&&^*P* < 0.01, and ^&&&^*P* < 0.001 versus the SHTL group, *n* = 3.

The above findings have shown that SHTL pretreatment upregulates the PPAR-*γ* pathway to decrease lipid accumulation in macrophages. To further validate the molecular mechanism of SHTL, GW9662, a specific PPAR antagonist, was used in this study. Given the observed inhibitory effect of SHTL on inflammation, we examined whether the anti-inflammatory role of SHTL was mediated by the activation of the PPAR-*γ* pathway. As shown in Figures 7(a) and 7(b), flow cytometric (FCM) analysis showed that SHTL reduced the ROS in LPS-induced mouse peritoneal macrophages, but the combination of SHTL and GW9662 has no obvious effect on the decrease of ROS levels by SHTL. Moreover, we found that the combination of SHTL and GW9662 reversed the inhibitory effects of SHTL on the levels of mRNA and protein levels of TNF-*α* induced by LPS stimulation (Figure 7(c)). Additionally, a similar trend was observed in the mRNA and protein levels of IL-6 in the LPS-induced macrophages, after combined treatment of SHTL with GW9662 (Figure 7(d)). These data suggested that SHTL inhibited LPS-induced inflammation in macrophages by the activation of the PPAR-*γ* signaling pathway.

### 3.8. SHTL Attenuates LPS- and Ox-LDL-Induced Lipid Accumulation through the Activation of the PPAR-*γ*/LXR-*α*/ABCA1 Pathway in Macrophages

**Figure 8:**
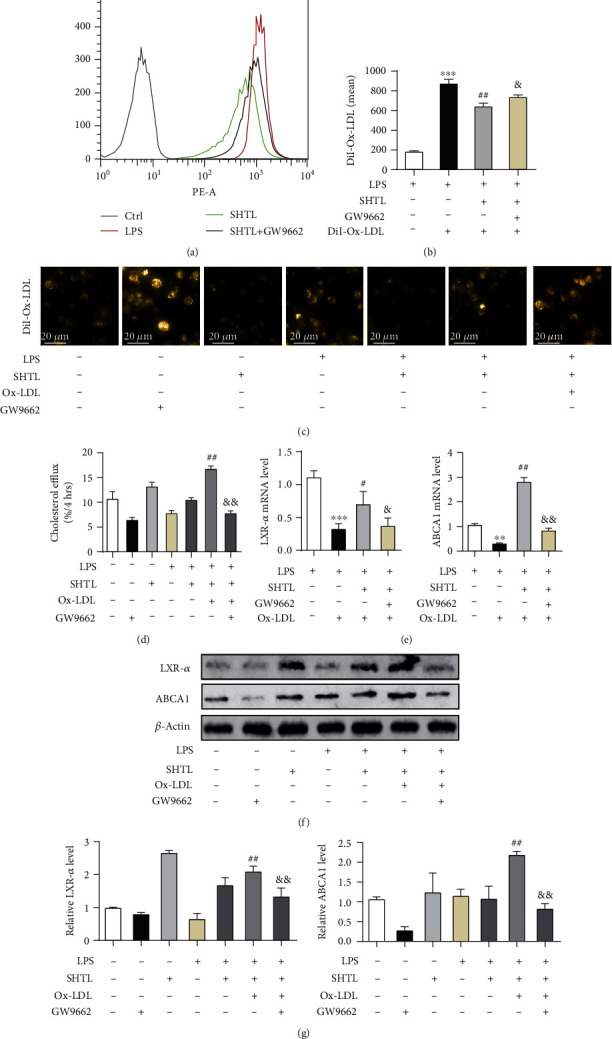
SHTL pretreatment attenuates LPS- and Ox-LDL-induced lipid accumulation via the activation of the PPAR-*γ*/LXR-*α*/ABCA1 pathway in mouse peritoneal macrophages. (a–c) After LPS incubation for 8 h, MPMs were treated with SHTL for 24 h prior to GW9662 treatment for 1 h. The lipid accumulation in MPMs was detected by DiI-Ox-LDL staining using flow cytometry and fluorescence microscopy imaging (scale bar = 20 *μ*m). The quantification of DiI-Ox-LDL fluorescence in different groups is shown. (d) THP-1-derived macrophages were pretreated as mentioned above. Cholesterol efflux was expressed as the percentage of fluorescence in the medium relative to total fluorescence. (e) The LXR-*α* and ABCA1 mRNA levels in LPS- and Ox-LDL-induced MPMs treated with SHTL and/or GW9662 for 24 h were detected by qPCR analysis. *β*-Actin was used for the normalization. (f, g) The protein levels of LXR-*α* and ABCA1 in macrophages from different groups were detected by Western blot analysis. Bar graphs show the relative expressions of LXR-*α* and ABCA1 from (f). *β*-Actin was the loading control. ^∗∗^*P* < 0.01 and ^∗∗∗^*P* < 0.001 versus the Ctrl group; ^#^*P* < 0.05 and ^##^*P* < 0.01 versus the Ox-LDL group; ^&^*P* < 0.05 and ^&&^*P* < 0.01 versus the SHTL group, *n* = 3.

Inflammatory- and lipid-accumulated macrophages play an important role in plaque formation in AS [37, 38]. Combined with the above results, the antiatherosclerosis effect of SHTL by the upregulation of the PPAR-*γ* pathway was further confirmed in a combined macrophage model. M1 proinflammatory macrophages were induced by LPS incubation followed by treatment with Ox-LDL or DiI-Ox-LDL to establish a lipid accumulation model. First, we found that LPS stimulation enhanced DiI-Ox-LDL accumulation in macrophages (Supplementary Figure S3). Consistent with the findings in Figure 3(b), SHTL decreased DiI-Ox-LDL accumulation in LPS-induced inflammatory macrophages, which was attenuated by GW9662 (Figures 8(a)–8(c)). The percentage of cholesterol efflux in DiI-Ox-LDL- and LPS-induced macrophages showed a similar trend in Figure 8(d). Moreover, the mRNA levels of LXR-*α* and ABCA1 were significantly decreased by incubation with LPS and Ox-LDL, which were increased by pretreatment with SHTL for 24 h. The combination of SHTL with GW9662 inhibited the increases in LXR-*α* and ABCA1 levels after SHTL treatment in the inflammatory and lipid overloading macrophages (Figure 8(e)). Also, Western blot analysis revealed a similar expression pattern of these key molecules, LXR-*α* and ABCA1 (Figures 8(f) and 8(g)). These results suggested that SHTL attenuated LPS- and Ox-LDL-induced lipid accumulation by the activation of the PPAR-*γ*/LXR-*α*/ABCA1 pathway in macrophages.

## 4. Discussion

Inflammatory response and lipid accumulation are regarded as the major pathogenesis of macrophage disorders in AS [39]. Therefore, the inhibition of inflammatory response and lipid accumulation in macrophages could slow down the process of AS [40, 41]. Here, we used network pharmacology to predict the potential targets of SHTL for protecting the macrophages. This study provides experimental evidence for the inhibitory effect of SHTL against inflammation response and lipid accumulation by activating the PPAR-*γ*/LXR-*α*/ABCA1 signaling pathway in macrophages and ApoE^−/−^ mice, which may be the potential mechanism of SHTL treatment for AS patients.

In AS, macrophages ensure tissue cholesterol homeostasis by removing Ox-LDL from the intercellular space [42]. LDL retained in the intima by binding to proteoglycan and was oxidatively modified to form lipid hydroperoxides, lysophospholipids, and other active moieties, which can induce the expression of adhesion molecules, chemokines, and proinflammatory cytokines in macrophages. Meanwhile, foamy macrophages are unable to induce an inflammatory response as strong as nonfoamy cells [43]. Importantly, TNF-*α*, IL-2, IFN-*γ*, and other proinflammatory cytokines downregulate cholesterol efflux and promote lipid accumulation in the lesion, which may accelerate macrophage transformation to foam cells and decrease cholesterol clearance [44]. Therefore, inflammation and lipid homeostasis are interacting in a dynamic equilibrium in macrophages during AS.

Most studies of TCM only focus on only one of them, like quercetin and rographolide, which have shown the functions of decreasing the level of ROS and the release of inflammatory cytokines by LPS in macrophages [45, 46]. In our study, we found that the pretreatment of SHTL showed similar efficacy of inflammatory inhibition to the above TCM. We also suggested that the pretreatment with SHTL could inhibit lipid accumulation in macrophages. Interestingly, SHTL pretreatment significantly inhibited the inflammation-induced foam formation. These results confirmed the therapeutic ability of SHTL in the multiple processes of AS.

Generally, it is difficult for us to explain the mechanisms involved in the TCM formulas because of the complicated compositions from multiple natural product-based prescriptions [47]. Network pharmacology can analyze multicomponent and multitarget agents to reveal the potential therapeutic mechanisms of TCM formulas [48]. In the present study, we combined the BATMAN-TCM, TCMSP, and STRING databases to find out the potential targets of SHTL [49]. We found that the PPAR pathway was the potential pathway of SHTL through enrichment analysis and identified its core targets using the STRING database analysis. Disease target network analysis showed that the PPAR-*γ*/LXR-*α*/ABCA1 pathway, the most valuable targets of SHTL, participated in inflammatory responses and cellular lipid homeostasis during AS, which was in line with our findings from LPS- and/or Ox-LDL-induced macrophage models. Furthermore, in HFD-induced ApoE^−/−^ mice, SHTL could reduce atherosclerotic plaque by upregulating the PPAR-*γ*/LXR-*α*/ABCA1 pathway. Finally, we used the selective PPAR-*γ* antagonist GW9662 to further verify the predicted pathway of SHTL by network pharmacology. Therefore, our study used several easy-to-operate databases and rescue experiments by a specific inhibitor to explore the targets of SHTL, the PPAR-*γ*/LXR-*α*/ABCA1 pathway, against inflammation and lipid accumulation [50].

As the common feature of TCM, SHTL contains multiple ingredients and has multiple intracellular targets. Although several major components of SHTL were identified by HPLC, the direct relationship between active ingredients of SHTL and their targets against AS needs to be further investigated. In the present study, the PPAR-*γ* signaling pathway was identified by network pharmacology and experimental outcome; other potential pathways of SHTL should be further detected. In addition, the protective role of SHTL for the treatment of AS needs to be evaluated in animal and clinical studies.

## 5. Conclusion

This study describes the protective effect of SHTL against inflammation and lipid accumulation in macrophages. The PPAR-*γ* pathway was predicted as the potential target of SHTL by network pharmacology and validated by a PPAR-*γ* antagonist, GW9662. These findings indicated that SHTL could protect macrophages by activating the PPAR-*γ*/LXR-*α*/ABCA1 pathway; our experiments in ApoE^−/−^ mice further support its antiatherosclerotic effect, which may provide a new insight into the mechanism of SHTL in the suppression of AS progression.

## Figures and Tables

**Table 1:** The compositions of the Shen-Hong-Tong-Luo (SHTL) formula.

Chinese name	Latin name	Family	Weight (g)	Part used	Voucher specimen
*Ren shen*	*Panax ginseng C*.*A*. *Mey*	Araliaceae	20	Root	180516-1
*Dan shen*	*Salvia miltiorrhiza Bunge*	Lamiaceae	25	Root	180913-1
*Hong jingtian*	*Rhodiola crenulata*	Crassulaceae	15	Root	180913-2
*Jin yinhua*	*Lonicera japonica Thunb*	Caprifoliaceae	15	Flower	180913-3
*Chi shao*	*Paeonia anomala* subsp. *veitchii* (*Lynch*) *D*.*Y*.*Hong & K*.*Y*.*Pan*	Ranunculaceae	15	Root	180913-4
*Gua lou*	*Trichosanthes kirilowii Maxim*	Cucurbitaceae	20	Fruit	180913-5
*Dang gui*	*Angelica sinensis* (*Oliv*.) *Diels*	Apiaceae	20	Root	180913-6
*Jiang xiang*	*Dalbergia odorifera T*.*C*.*Chen*	Leguminosae	10	Rhizome	180913-7

**Table 2:** The main components of the Shen-Hong-Tong-Luo (STHL) formula predicted by network pharmacology.

Herbs	Number	Components
*Panax ginseng C*.*A*. *Mey*	155	Ginsenoyne B, ginsenoyne E, argininyl-fructosyl-glucose uridine alpha-cadinol, octanal, beta-elemene, chrysanthemaxanthin, biotin, chikusetsusaponin IV, pandamine, panasinsanol A, N-pentadecane, ginsenoside F1, aposiopolamine, D-mannuronic acid, palmitic acid, etc.
*Salvia miltiorrhiza Bunge*	75	Miltionone II, miltipolone, danshenol B, tanshiquinone B, miltirone, isocucurbitacin D, baicalin, tigogenin neotigogenin, isotanshinone I, monomethyl lithospermate, salonitenolide, magnesium lithospermate B, miltionone I, etc.
*Rhodiola crenulata*	1	Salidroside
*Lonicera japonica Thunb*	72	Menthyl acetate, inositol, 2-furaldehyde, linalyl oxide, carvacrol, methyl palmitate, methyl linoleate, chrysoeriol, eugenol methyl ether, farnesyl acetate, loganoside, benzyl cyanide, lonicerin, 2-heptadecanone, loganin, macrocarpal A, stigmasterol, geranyl acetate, citronellyl acetate, etc.
*Paeonia anomala* subsp. *veitchii*	31	Trichothecin, menthyl acetate, tetradecane, karounidiol 3-benzoate, methyl palmitate, myristicin, N-nonanol, palmitone, gamma-aminobutyric acid, cucurbitacin B, naphthalene, 5, 25-stigmastadien-3beta-Ol-beta-D-glucoside, karounidiol, caproic acid, stigmastanol, stearin, 20-hexadecanoylingenol, bryonolic acid, etc.
*Trichosanthes kirilowii Maxim*	19	Oxypaeoniflorin, lactiflorin, paeonin, benzoylpaeoniflorin, albiflorin, paeonolide, galloylpaeoniflorin, (+)-catechin, gallocatechin, paeoniflorin, (-)-catechin, beta-sitosterol, acetic acid, paeonoside, paeonol, paeoniflorigenone, catechin, epigallocatechin, daucosterol, etc.
*Angelica sinensis* (*Oliv*.) *Diels*	120	Dimethyl phthalate, suchilactone, beta-myrcene, ethanol, tetradecane, carvacrol, 2,4,5-trimethylbenzaldehyde, 2,4-dimethylbenzaldehyde, 2′,4′-dihydroxyacetophenone, uridine, 2-propene, 3-O-tetradecanoyl-1-cyano-2-methyl-1, beta-elemene, chrysanthemaxanthin, palmitic acid, azelaic acid, dimethyl-beta-propiothetin, 4-ethylresorcinol, vanillin, dimethyl sebacate, etc.
*Dalbergia odorifera T*.*C*.*Chen*	4	Nordalbergin, isodalbergin, dalbergin, luteolin

## Data Availability

The data used to support the findings of this study are available from the corresponding authors upon request.

## Abbreviations

AS: Atherosclerosis
SHTL: Shen-Hong-Tong-Luo
Ox-LDL: Oxidized low-density lipoprotein
PPAR-*γ*: Peroxisome proliferator-activated receptor-*γ*
LXR-*α*: Liver X receptor-*α*
ABCA1: ATP-binding cassette subfamily A member 1
TCM: Traditional Chinese medicine
LPS: Lipopolysaccharide
DiI-Ox-LDL: DiI-labeled Ox-LDL
HFD: High-fat diet.

## Supplementary Materials

Supplementary Materials Supplementary Figure S1 is a supplement to the result The Target Prediction of SHTL by Network Pharmacology; the disease target network verified the central role of PPAR-*γ* in regulating AS, inflammation, cardiovascular disease, hypertension, and other diseases. Supplementary Figure S1: the disease target network of SHTL was shown by Gephi. The bigger circles represented the disease, and the smaller circles represented the predicted targets. Supplementary Figure S2 is a supplement to the result SHTL Activates the PPAR-γ/LXR-α/ABCA1 Pathway in the Ox-LDL-Induced Macrophages. Supplementary Figure S2: the effect of Ox-LDL induction and SHTL treatment on ABCA1 and LXR-*α* mRNA levels in MPMs. (A) Mouse peritoneal macrophages (MPMs) were stimulated without or with Ox-LDL (40 *μ*g/mL). The time-dependent effect of Ox-LDL in MPMs was evaluated using qPCR analysis after the normalization of *β*-actin. (B) MPMs were incubated with SHTL for 6 h, 12 h, and 24 h. The time-dependent effect of SHTL in MPMs was evaluated using qPCR analysis after the normalization of *β*-actin. Supplementary Figure S3 is a supplementary support to the result SHTL Attenuates LPS- and Ox-LDL-Induced Lipid Accumulation through the Activation of the PPAR-γ/LXR-α/ABCA1 Pathway in Macrophages. Supplementary Figure S3: the effect of DiI-Ox-LDL or combined with LPS on lipid accumulation in MPMs. After LPS incubation for 8 h, the lipid accumulation in MPMs was detected by DiI-Ox-LDL staining using flow cytometry. The blue line was the DiI-Ox-LDL group and the red line was LPS+DiI-Ox-LDL group. Supplementary Table 1 is the HPLC gradient elution process. Supplementary Table 2 is the sequences of primers used in the qRT-PCR. Supplementary Table 3 is the top 20 enrichment pathways of the Shen-Hong-Tong-Luo formula. It is the raw data for Figure 4(b). Term description was given for each name of the top 20 pathways. *P* value, Benjamini value, coverage, and enrich ratio were predictive attributes of each pathway. Supplementary Table 4 is the significant enrichment of diseases and their targets that belong to the Shen-Hong-Tong-Luo formula.
