# Supplementary material for: Shen-Hong-Tong-Luo Formula Attenuates Macrophage Inflammation and Lipid Accumulation through the Activation of the PPAR-γ/LXR-α/ABCA1 Pathway
Source: Oxid Med Cell Longev. 2020 Oct 5;2020:3426925. doi: 10.1155/2020/3426925 (PMC7556105; doi:10.1155/2020/3426925)
Supplement: Supplementary Materials — Supplementary Figure S1 is a supplement to the result The Target Prediction of SHTL by Network Pharmacology; the disease target network verified the central role of PPAR-γ in regulating AS, inflammation, cardiovascular disease, hypertension, and other diseases. Supplementary Figure S1: the disease target network of SHTL was shown by Gephi. The bigger circles represented the disease, and the smaller circles represented the predicted targets. Supplementary Figure S2 is a supplement to the result SHTL Activates the PPAR-γ/LXR-α/ABCA1 Pathway in the Ox-LDL-Induced Macrophages. Supplementary Figure S2: the effect of Ox-LDL induction and SHTL treatment on ABCA1 and LXR-α mRNA levels in MPMs. (A) Mouse peritoneal macrophages (MPMs) were stimulated without or with Ox-LDL (40 μg/mL). The time-dependent effect of Ox-LDL in MPMs was evaluated using qPCR analysis after the normalization of β-actin. (B) MPMs were incubated with SHTL for 6 h, 12 h, and 24 h. The time-dependent effect of SHTL in MPMs was evaluated using qPCR analysis after the normalization of β-actin. Supplementary Figure S3 is a supplementary support to the result SHTL Attenuates LPS- and Ox-LDL-Induced Lipid Accumulation through the Activation of the PPAR-γ/LXR-α/ABCA1 Pathway in Macrophages. Supplementary Figure S3: the effect of DiI-Ox-LDL or combined with LPS on lipid accumulation in MPMs. After LPS incubation for 8 h, the lipid accumulation in MPMs was detected by DiI-Ox-LDL staining using flow cytometry. The blue line was the DiI-Ox-LDL group and the red line was LPS+DiI-Ox-LDL group. Supplementary Table 1 is the HPLC gradient elution process. Supplementary Table 2 is the sequences of primers used in the qRT-PCR. Supplementary Table 3 is the top 20 enrichment pathways of the Shen-Hong-Tong-Luo formula. It is the raw data for Figure 4(b). Term description was given for each name of the top 20 pathways. P value, Benjamini value, coverage, and enrich ratio were predictive attributes of each pathway. Supplemen [file 3426925.f1.pdf]

Supplementary information

Shen Hong Tong Luo formula attenuates macrophage inflammation and lipid accumulation through the activation of PPAR-γ/LXR-α/ABCA1 pathway

Zepeng Zhang[a,b], Lu Zhai[a,b], Jing Lu[a,b], Sanmiao Sun[c], Dandan Wang[a,b], Daqing Zhao[b,d], Liwei Sun[a,b], Weimin Zhao[e], Xiangyan Li[b,d*], Ying Chen[f*]

[a]Research Center of Traditional Chinese Medicine, the Affiliated Hospital to Changchun University of Chinese Medicine, Changchun, Jilin, China

[b]Jilin Provincial Key Laboratory of BioMacromolecules of Chinese Medicine, Changchun University of Chinese Medicine, Changchun, Jilin, China

[c]College of Traditional Chinese Medicine, Changchun University of Chinese Medicine, Changchun, Jilin, China

[d]Jilin Ginseng Academy, Changchun University of Chinese Medicine, Changchun, Jilin, China

[e]Center of Preventive Treatment of Disease, the Affiliated Hospital to Changchun University of Chinese Medicine, Changchun, Jilin, China

[f]Department of Cardiology, the Affiliated Hospital to Changchun University of Chinese Medicine, Changchun, Jilin, China

*Corresponding author: Xiangyan Li, Jilin Provincial Key Laboratory of BioMacromolecules of Chinese Medicine, Jilin Ginseng Academy, Changchun University of Chinese Medicine, Changchun, Jilin, China. Tel.: +86-431-86177630, Fax: +86-431-86177630, E-mail: xiangyan_li1981@163.com. Ying Chen, Department of Cardiology, the Affiliated Hospital to Changchun University of Chinese Medicine, Changchun, Jilin, China. Tel.: +86-431-86177226, Fax: +86-431-86177226, E-mail: chenying66323@163.com.

Supplementary Figure S1

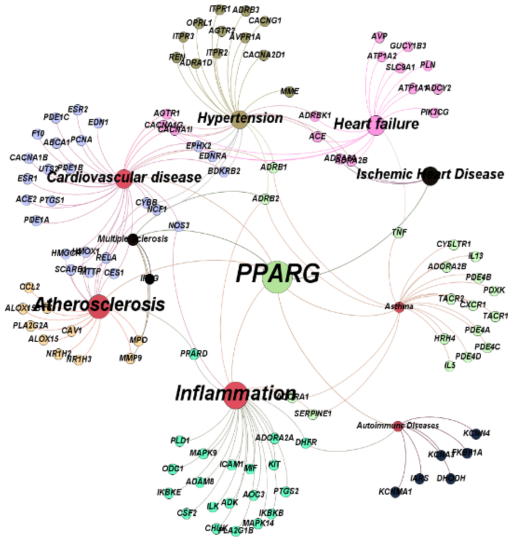

The disease-target network of SHTL was shown by Gephi. The bigger circles represented the disease and the smaller circles represented the predicted targets.

Supplementary Figure S2

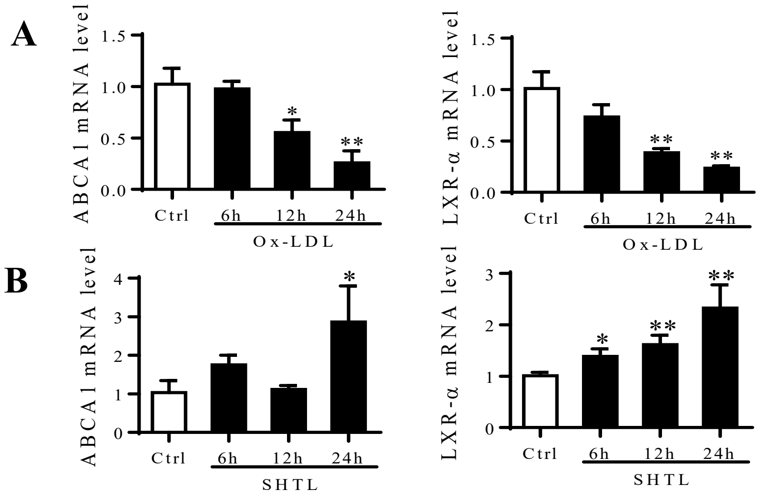

(A) Mouse peritoneal macrophages (MPMs) were stimulated without or with Ox-LDL (40μg/mL). The time-dependent effect of Ox-LDL in MPMs was evaluated using qPCR analysis after the normalization of β-Actin. **(B)** MPMs were incubated with SHTL for 6 h, 12 h and 24 h. The time-dependent effect of SHTL in MPMs was evaluated using qPCR analysis after the normalization of β-Actin.

Supplementary Figure S3

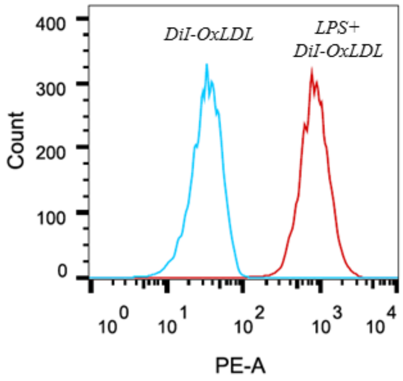

After LPS incubation for 8 h, the lipid accumulation in MPMs was detected by DiI-Ox-LDL staining using flow cytometry.

Supplementary Table 1 HPLC gradient elution process

Time(min)	Mobile phase A	Mobile phase B	Time(min)	Mobile phase A	Mobile phase B
0-20	10~18%	90~82%	65~85	32%	68%
20-25	18~20%	82~80%	85~95	32~90%	68~10%
25~45	20~30%	80~70%	105~110	10%	90%
45~60	30%	70%			

Supplementary Table 2 Sequences of primers used in the qRT-PCR.

Primer		Sequence
PPAR-γ	forward	GCAGCTACGCTCCTTTCTTG
	reverse	TGTTGCCCCTCTTTAGTGTCA
LXR-α	forward	ATCGCCTTGCTGAAGACCTCTG
	reverse	GATGGGGTTGATGAACTCCACC
ABCA1	forward	GCTTGTTGGCCTCAGTTAAGG
	reverse	GTAGCTCAGGCGTACAGAGAT
IL-6	forward	CCAAGAGGTGAGTGCTTCCC
	reverse	CTGTTGTTCAGACTCTCTCCCT
TNF-α	forward	CCCTCACACTCAGATCATCTTCT
	reverse	GCTACGACGTGGGCTACAG
β-Actin	forward	GGCTGTATTCCCCTCCATCG
	reverse	GCTACGACGTGGGCTACAG

Supplementary Table 3 The Top20 pathway Enrichment of Shen Hong Tong Luo formula

Term description	P-value	Benjamini-value	Coverage	EnrichRatio
beta-Alanine metabolism	1.12E-05	9.00E-05	17	2.8
Serotonergic synapse	2.86E-06	2.38E-05	44	1.9
PPAR signaling pathway	2.61E-06	2.25E-05	31	2.2
Alanine, aspartate and glutamate metabolism	5.23E-07	4.67E-06	21	2.9
TGF-beta signaling pathway	3.90E-07	3.76E-06	36	2.3
Tryptophan metabolism	2.14E-07	2.15E-06	23	2.8
Propanoate metabolism	2.01E-07	2.11E-06	20	3.1
Cysteine and methionine metabolism	1.74E-07	1.91E-06	22	2.9
Cardiac muscle contraction	3.20E-08	3.86E-07	37	2.3
cGMP - PKG signaling pathway	1.25E-08	1.59E-07	65	1.9
Biosynthesis of amino acids	5.05E-09	7.16E-08	37	2.5
Purine metabolism	1.48E-09	2.23E-08	68	2
Phenylalanine metabolism	7.02E-10	1.13E-08	16	4.4
Glycine, serine and threonine metabolism	3.52E-10	6.06E-09	26	3.3
Valine, leucine and isoleucine degradation	5.05E-11	9.36E-10	29	3.3
Tyrosine metabolism	4.15E-11	8.33E-10	27	3.4
Carbon metabolism	1.09E-12	2.63E-11	54	2.6
Arginine and proline metabolism	2.57E-16	1.03E-14	41	3.4
Calcium signaling pathway	2.94E-22	2.36E-20	94	2.6
Neuroactive ligand-receptor interaction	7.81E-37	9.41E-35	148	2.7

Supplementary Table 4 Significant enrichment of diseases and their targets belong to Shen Hong Tong Luo formula

Disease	Target
Atherosclerosis	ABCA1, ADIPOQ, CREB1, ROCK1, SCARB1, ALOX15, ALOX15B, CAV1, CCL2, CES1, HMGCR, HMOX1, MMP9, MPO, NR1H2, NR1H3, PLA2G2A, PPARD, PPARG
Inflammation	ADAM8, ADK, ADORA1 ,ADORA2A, AOC3, CHUK, DHFR, ICAM1, IKBKB, IKBKE, ILK, KIT, MAPK14, MAPK9, NOS3, ODC1, PLA2G1B, PLD1, PPARD, PPARG, PTGS2, SERPINE1
Cardiovascular disease	ABCA1, ADRB1, AGTR1, BDKRB2, CACNA1B, CES1, EDN1, EDNRA, EPHX2, ESR1, ESR2, F10, HMGCR, , HMOX1, NOS3, PCNA, PDE1B, PTGS1, SCARB1, SCD, UTS2
Myocardial infarction	BDKRB2, HMGCR, NPR2, PIK3CG, PTGS2, UTS2R
Hypertension	ACE, ADRA1D, ADRA2A, ADRA2B, ADRB1, ADRBK1, AGTR1, AGTR2, AVPR1A, BDKRB2, CACNA2D1, CACNG1, EDNRA, EPHX2, MME, OPRL1
Heart failure	ACE, ADCY2, ADRA2A, ADRA2B, ADRBK1, AGTR1, ATP1A1, ATP1A2, AVP, GUCY1B3, PIK3CG, PLN, SLC9A1, TNF
